# Evaluation of apical preparations performed with ultrasonic diamond and stainless steel tips at different intensities using a scanning electron microscope in endodontic surgery

**DOI:** 10.4317/medoral.17961

**Published:** 2012-08-28

**Authors:** Ramón Rodríguez-Martos, Daniel Torres-Lagares, Lizett Castellanos-Cosano, María A. Serrera-Figallo, Juan J. Segura-Egea, Jose L. Gutierrez-Perez

**Affiliations:** 1 Department of oral surgery. Faculty of dentistry. University of Seville; 2 Department of dental pathology and endodontic. Faculty of dentistry. University of seville; 3 ….

## Abstract

Objective: The objective of our study is to analyse (with the help of scanning electron microscopes) the quality of the dental root surface and the appearance of dental cracks after performing apical preparations using two diffe-rent types of ultrasonic tips. Study design: We used 32 single-rooted teeth that underwent a root canal and apical resection. Afterwards, the teeth were divided into 4 groups of 8 teeth each, with preparations of the apical cavities in the following manner: Group 1: stainless steel ultrasonic tip at 33KHz. Group 2: stainless steel ultrasonic tip at 30KHz. Group 3: diamond ultrasonic tip at 30KHz. Group 4: diamond ultrasonic tip at 33 KHz. The quality of the root surface and the presence of cracks were evaluated by one single observer using a scanning electron microscope. Results: All of the teeth in our study had cracks after the apical preparations. The mean number of cracks per tooth ranged between 6.1±1.9 (group 1) and 3.5±2.4 (group 4), with a significantly higher number found in the groups that used stainless steel tips (P=.03). The types of cracks produced involved: 8 complete cracks (4.5%), 167 incomplete cracks (94.4%), and 2 intradentinal cracks (1.1%), with no significant differences observed between the different frequencies used for each group. Conclusions: Stainless steel ultrasonic tips provoked a larger number of cracks than diamond tips. The frequency of vibration used did not have any effect on the number of cracks found.

** Key words:**Apicoectomy, scanning electron microscope, therapeutic ultrasound, endodontic surgery, dentinal crack.

## Introduction

Endodontic surgery is a surgical procedure that consists of eliminating pathological periapical tissue, excision of the root surface (including apical accessory canals), and finally a sealant or closing of the root canal or canals against the entry of pathogens, thus reaching the objective of creating optimal conditions for health, tissue regeneration, and the formation of a new support structure for the tooth.

The ideal apical preparation should comply with a series of requirements: walls parallel to the longitudinal axis of the tooth, 3mm depth, and central location with respect to the root (1).

However, given the complexity of the system of root canals, the location of the roots, the rigidity of hand instruments, etc., reaching these ideal characteristics using traditional rotary techniques had become practically impossible (2).

In the early nineties, several different ultrasonic points appeared on the market that were specially designed for apical preparations (3-6). The advantages of ultrasonic instruments can be summarised in the following points: they allow us to follow the longitudinal axis of the tooth, while conserving the morphology of the root canal; the apical cavities conform much easier, safer, and with greater precision, the level of the cut obtained in the root resection is near perpendicular to the longitudinal axis of the tooth, reducing the number of dentinal tubules exposed on the root surface and minimising apical leakage, the cavities are smaller and more centrally located, reducing the risk of root perforation, and finally better cleaning of the cavity walls, reducing the volume of dentinal residues (7-9).

However, and despite the excellent results obtained using ultrasonic points, it has been shown that this technique is not without its problems, such as the appearance of cracks during the apical preparation (10). These microfractures could influence the healing process around the root, and could lead to failure due to microleakage (11-14). Several different studies have analysed the effects that these ultrasonic instruments have on the root surface during endodontic surgical procedures, using light microscopes, scanning electron microscopes (15), and endoscopy (16).

The objective of our study was to analyse (using a scanning electron microscope) the quality of the root surface and the appear-ance of cracks following apical preparations (in extracted single-rooted teeth) using diamond and stainless steel tips at different intensities of vibration frequency.

## Material and Methods

In our study, we used 32 single-rooted teeth with single canals. All teeth were alive, having been removed for orthodontic and/or periodontal reasons from individuals of 18-50 years of age. The study complied with the Helsinki ethical guidelines and was approved by the ethics committee of the University of Seville.

We selected teeth that had not been restored, with intact roots and mature apexes. Following extraction, the soft tissue was debrided manually from the root surface using periodontal curettes. The teeth were cleaned and placed in a 5% sodium hypochlorite solution during 30 minutes, cleaned again using saline solution, and immediately placed in 5% formaldehyde during 24 hours.

We gained access into the pulp canals using 014 round tungsten carbide burs and endo Z burs (Dentsply International, York, USA), and set the working lengths at 0.5mm from the apical foramen using No. 15 K-files (DentsplyMaillefer, Ballaigues, Switzerland). We took x-rays of all teeth with the file in place in order to ensure the working length and to exclude any canals with irregular anatomy. We worked the canals using a step-back manual technique until reaching a No. 35 file size, irrigating with 2.5% sodium hypochlorite. Afterwards, the canals were dried and obturated using the lateral condensation technique (A 022E guttapercha, DentsplyMaillefer. Ballaigues, Switzerland) and AH PLUS cement (Dentsply. York, USA). The opening to the chamber was sealed with glass ionomer cement and the teeth were stored at 37ºC and 100% humidity in order to complete the sealing process.

We then used a diamond disc saw (Diamond Saw Blade, Buehler. Illinois, USA) mounted on a precision cutter (Isomet Low Saw, Buehler, Illinois, USA), with constant irrigation, to make a cross-sectional cut through each of the 32 teeth three millimetres from the apex, forming a 90º angle with the longitudinal axis of the tooth. After the cut was made, we examined each of the teeth using a stereoscopic microscope (Leica MZ16 with 16:1 zoom) at X2, X4, and X8 magnifications in order to detect fractures that may have been provoked by the root resection before preparing the apical cavities using ultrasonic tips.

The teeth were then divided into 4 groups of 8 teeth each. The apical cavities were prepared using a SatelecSuprasson P5 Booster ultrasonic device (Satelec, Paris, France), with constant irrigation, for 20 seconds.

• In group 1, we used a stainless steel tip at maximum frequency (33KHz)

• In group 2, we used a stainless steel tip at medium frequency (30KHz)

• In group 3, we used a diamond tip at medium frequency (30KHz)

• In group 4, we used a diamond tip at maximum frequency (33KHz).

In each group, two different new ultrasonic tips were used, that is to say, one tip for the first 4 teeth and another for the other 4 in each group.

The next step was a preparation of the samples for analysis using a scanning electron microscope (SEM Jeol 6460LV, Jeol, Tokyo, Japan), in accordance with the direct method proposed by Janda for research and analysis of natural teeth using a scanning electron microscope (17). After dehydrating and drying the teeth, the samples were coated in gold for examination under the microscope at X20 and X70 magnifications.

Cracks were defined as lines or faults that appeared in the dentinal surface and that appeared to interrupt the integrity of the dentine (18). We quantified the number of cracks that were found in each group, categorising the cracks according to the classification system proposed by Beling and colleagues (19):

• Complete cracks: extend from the root canal to the external surface of the root.

• Incomplete cracks: extend from the root canal towards the external surface of the root at a varying distance, but without reaching it completely.

• Intradentinal cracks: appear to advance in the vestibular-lingual or mesial-distal region within the root canal.

Finally, we analysed the quality of the preparation margin following this classification scheme (20):

• Type A margin. Ideal preparation, no defects

• Type B margin. Isolated defects.

• Type C margin. Irregular, worn margins.

• Type D margin. Worn margins with defects from the ultrasonic tip.

The minimum sample size was calculated for the comparison of two independent means using Query Advisor ® (Version 7.0). We entered the data into a Microsoft Excel 2007 spreadsheet® (Microsoft Corporation. Washington. USA) and performed all descriptive statistical analyses using SPSS version 11 (® SPSS Inc., Chicago, USA).

We used the Kruskall-Wallis H test for multiple comparisons of continuous variables to compare the results between groups, and the Mann-Whitney U test to compare between two variables. We used the chi-square (χ²) test to compare qualitative variables.

## Results

X-ray imaging revealed that all roots were prepared and obturated to the appropriate depth. None of the samples had to be excluded because of improper obturation technique. After the apical resection, we did not observe fracturing or changes to the preparation margins.

**Figure 1:**
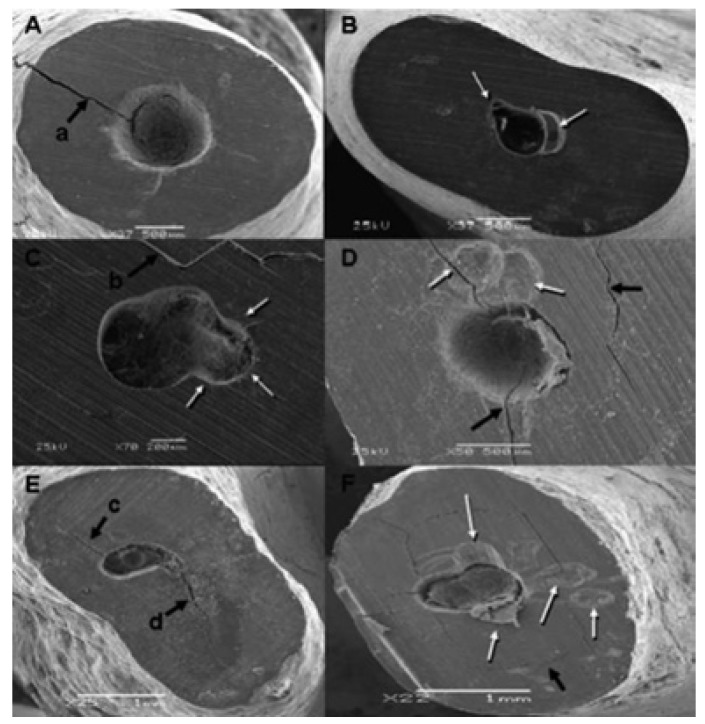
A) Example of the root apex with a complete crack (a, black arrow). B) Example of the root apex with a type C defect (white arrows), the example was from a tooth in group 4. C) Root apex with a dentinal crack (b, black arrow) and type B margin. Here we can observe a margin defect possibly caused by the contact between the angled portion of the tip and the margin of the cavity. The example belongs to group 4. D) Root apex with several cracks and a type C surface (white arrows). The tooth was in group 1. E) Root apex with an incomplete crack (c, black arrow), from group 3. F) Root apex with a complete crack, several incomplete cracks, and an intradentinal crack (black arrow). The margin was evaluated as type D, and the tooth was from group 1.

Scanning electron microscope analysis.- All samples from all groups had one or more cracks. The total number of cracks was 177 ( Table 1). Group 1 had the highest number of cracks with 49, whereas group 3 had the lowest number, at 39. The number of cracks observed was significantly higher in groups 1 and 2 (stainless steel tips) than groups 3 and 4 (diamond tips) (P=.03). However, there were no differences between groups in terms of vibration frequency (P>.05). The mean number of cracks per tooth ranged between 6.1±1.9 (group 1) and 3.5±2.4 (group 4). We did not observe any correlation between the number of cracks and the frequency of vibration used (P=.48). However, the groups with stainless steel tips had more cracks than the groups with diamond tips (P=.03). We observed no differences between groups in terms of the type of cracks observed (P>.05). A total of 8 teeth (4%) had complete cracks (Fig. 1). Incomplete cracks were the most commonly observed in all groups (94%) (Fig. 1). We only observed two intradentinal cracks (1%), all in group 1 (Fig. 1).

**Table 1:**
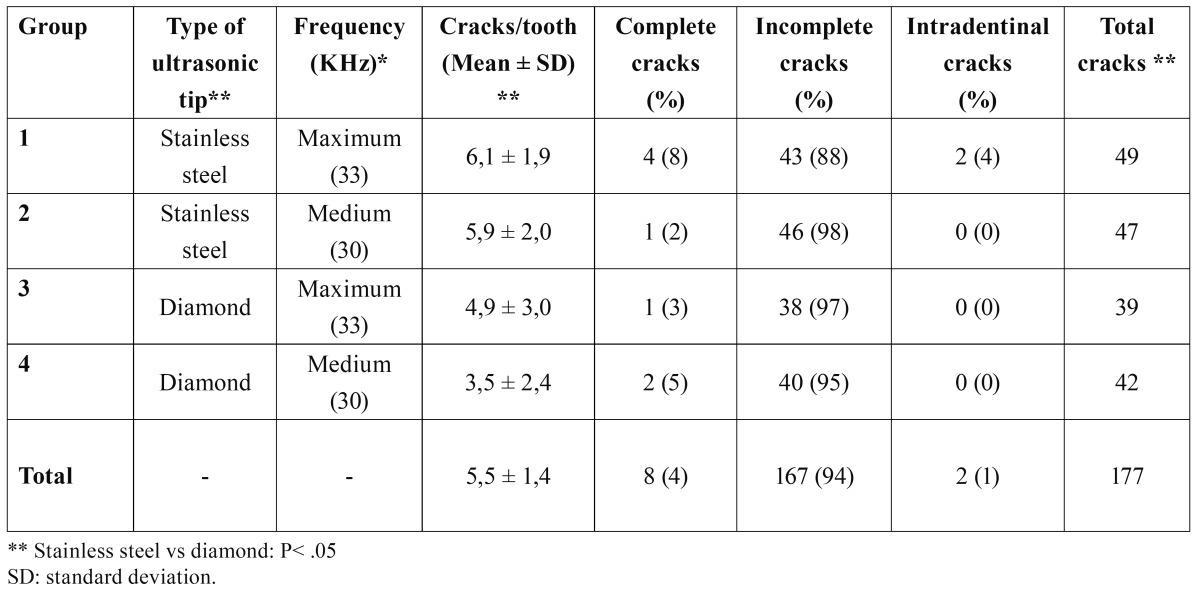
Results of the scanning electron microscope evaluation: number and type of cracks.

All samples had some type of margin defect in the qua-lity analysis of the preparation margins and root surface ( Table 2). We found no relationship between the margin quality and the type of tip or the vibration frequency used (P>.05). In all groups, over half of all samples had some type of visible defect (type B) (Fig. 1). Type C margins were observed in 9 teeth (28%) (Fig. 1). We found type D margins in 18.8% of all teeth (Fig. 1).

**Table 2:**
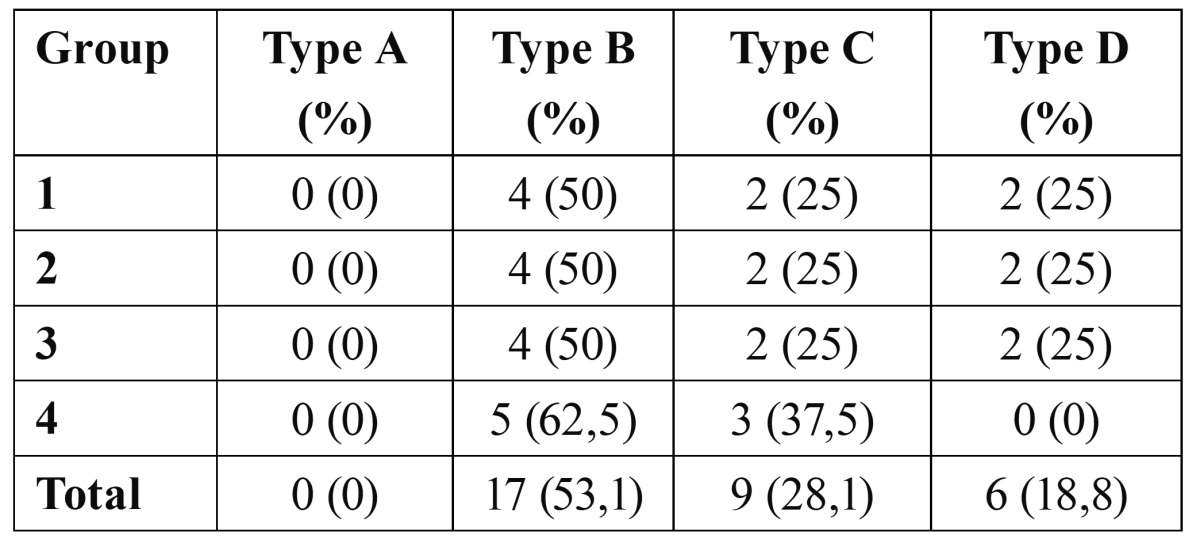
Results from the scanning electron microscope analysis: cavity margin quality.

## Discussion

Apical preparations using the traditional technique were performed using rotary tools (hand instruments and tungsten carbide burs). These instruments required a certain angle of entry for the apical resection that could jeopardise the root remnant, reducing the corona/root proportion and affecting the periodontium (21). The introduction of ultrasonic tips in endodontic surgery has facilitated a better treatment of the root apex (13,15). However, despite the excellent results obtained using ultrasonic tips, there has also been an elevated incidence of cracks detected when apical preparations are performed using these tips (2,15,19).

Our study used the roots of extracted teeth to analyse the surface quality and appearance of dentinal cracks following apical preparations using ultrasonic tips. The scanning electron microscope analysis demonstrated that stainless steel tips caused a greater number of cracks than diamond tips. On the other hand, the frequency used for the ultrasonic device had no influence on the number of cracks produced.

The results from this in vitro study (along with other publications) should be interpreted while taking into account that these studies do have some inherent limitations related to the appearance of artefacts in the samples: processes of apical resection and cavity preparation (22), lack of a periodontal ligament during the instrumentation procedure and dentine dehydration (23), and the stress suffered by the tooth upon extraction, during manipulation, and upon preservation all could predispose the tooth to cracks (24). In this study, all teeth analysed had dentinal cracks. Since the duration of the preparation was not limited, this could mean that this is one factor that contributed to the appearance of cracks.

As regards the types of cracks produced, 94.4% were incomplete cracks, 4.5% were complete cracks, and only 1.1% were intradentinal cracks. Few studies have analysed the different types of cracks produced following apical preparations using ultrasonic instruments (25). Rainwater and colleagues (26) compared stainless steel and diamond tips using low-frequency ultrasound and found no significant differences in the number or type of cracks produced. Other studies have detected intradentinal cracks more than any other type (19). We observed no statistically significant differences among the different groups in our study in terms of margin quality, coinciding with the results from previous studies (26). Our results show that stainless steel tips provoke a greater number of cracks than diamond tips. On the other hand, other authors have found no differences in the cracks produced between these two types of tips (27). Still other authors have shown that diamond tips produce excellent results in apical preparations, superior to those achieved using stainless steel tips. Diamond tips were introduced with the goal of reducing dentinal fractures along with their capacity to eliminate dentine more rapidly, reducing the amount of time needed for the instrument to be in contact with the root surface (28).

Several different studies have researched the effect that changing the frequency of the ultrasonic device has on the root surface, with controversial results (29). In our study, we found no relationship between ultrasonic frequency and the number of cracks, coinciding with the results from other publications (30). On the other hand, when analysing the cutting capacity of two different ultrasonic devices with two different tips and maximum and medium frequencies, all variables (ultrasonic device, frequency, and type of tip) appeared to affect the cutting capacity (28). The vibrating power of the ultrasonic unit does appear to have a relationship with the appearance of cracks, based on previously published studies (28).

The clinical importance of dentinal cracks provoked by apical surgeries is not completely clear, although complete cracks appear to favour leakage and promote recurrent apical infections (16). As such, if the alterations provoked by ultrasonic tips in the root apex influence clinical results, our efforts should be focused on minimising the appearance of these alterations, such as cracks.

In summary, we can conclude that stainless steel ultrasonic tips provoke a larger number of cracks than diamond tips. The selected frequency of vibration appears to not have any relationship with the number of cracks produced.
